# Effects of the Chinese herbal medicine Hong Huang decoction, on myocardial injury in breast cancer patients who underwent anthracycline-based chemotherapy

**DOI:** 10.3389/fcvm.2022.921753

**Published:** 2022-07-22

**Authors:** Sihan Cao, Jingxian Xue, Lu Chen, Yun Hao, Meijuan Lu, Ming Feng, Huanhuan Wang, Jun Zhou, Chang Yao

**Affiliations:** ^1^Department of Breast Disease, Affiliated Hospital of Nanjing University of Chinese Medicine, Nanjing, China; ^2^The First Clinical College, Nanjing University of Chinese Medicine, Nanjing, China; ^3^Department of Echocardiography, Affiliated Hospital of Nanjing University of Chinese Medicine, Nanjing, China

**Keywords:** breast cancer, chemotherapy, anthracycline, Hong Huang decoction, GLS

## Abstract

**Objective:**

To assess the effects of Hong Huang Decoction (HHD), a Chinese herbal medicine, on myocardial injury in breast cancer patients who underwent anthracycline (ANT)-based chemotherapy.

**Methods:**

A total of 51 patients with breast cancer who underwent an ANT-based chemotherapy program and met the inclusion/exclusion criteria were allocated to the treatment or placebo groups using a random number generation process. Patients in the treatment group received liquid HHD twice a day. Treatment was given from 1 day prior to chemotherapy up to the end of chemotherapy (after 6 months). Participants in the placebo group received a placebo over the same schedule. Left ventricular ejection fraction (LVEF), global longitudinal strain (GLS), diagnostic markers of acute myocardial infarction [e.g., lactate dehydrogenase (LDH), creatine kinase-MB (CK-MB), and B-type natriuretic peptide (BNP)], nitric oxide (NO), superoxide dismutase (SOD), as well as pro-inflammatory cytokines [e.g., tumor necrosis factor-α (TNF-α), interleukin-6 (IL-6), and human C-reactive protein (CRP)], and anti-inflammatory cytokine interleukin-10 (IL-10), were outcome measures assessed before chemotherapy, 3 and 6 months after chemotherapy.

**Results:**

Compared to the placebo group, the GLS value was significantly higher in the treatment group (19.95 ± 1.16 vs. 19.06 ± 1.64, *P* ≤ 0.001). Significant differences were also noted for levels of SOD (689.71 ± 203.60 vs. 807.88 ± 182.10, *P* < 0.05), IL-6 (58.04 ± 22.06 vs. 194.20 ± 40.14, *P* ≤ 0.001), IL-10 (237.90 ± 94.98 vs. 68.81 ± 32.92, *P* ≤ 0.001), NO (75.05 ± 26.39 vs. 55.83 ± 19.37, *P* ≤ 0.005), and TNF-α (301.80 ± 134.20 vs. 680.30 ± 199.60, *P* ≤ 0.001) in the patients before chemotherapy compared to 6 months after initiating chemotherapy.

**Conclusion:**

HHD regulated the levels of IL-6, IL-10, SOD, NO, and TNF-α. The results demonstrated that GLS is a better indicator of early myocardial injury compared to LVEF, and HHD could modulate oxidative stress to protect against ANT cardio toxicity.

**Clinical trial registration:**

Chinese Clinical Trial Registry, identifier ChiCTR1900022394. Date of registration: 2019-04-09.

## Introduction

Breast cancer is one of the leading causes of morbidity and mortality worldwide (1). Advances in early diagnosis and treatment of breast cancer have contributed to the steady increase in the number of cancer survivors. Nonetheless, such effective cancer therapies have led to a noticeable increase in cardiovascular complications in a significant proportion of cancer survivors (2). Furthermore, cardiovascular complications have become the most common cause of death in breast cancer patients (3). As such, during comprehensive treatment of breast cancer, cardiotoxicity resulting from the chemotherapy agents and targeted therapies are worthy of further investigation. Anthracyclines (ANTs), such as daunorubicin, doxorubicin, idarubicin, and epirubicin, are chemotherapeutic agents used to treat diverse types of cancer (4). Oxidative stress plays an important role in cardiotoxicity induced by antineoplastic drugs and participates in toxic reactions (5). The mechanisms underlying ANT-induced cardiotoxicity are complex, multifactorial, may involve genetic mutations and continue to be a mystery. The most well-known mechanism is the generation and accumulation of reactive oxygen species (ROS) and reactive nitrogen species (RNS) that account for lipid peroxidation and DNA damage in cardiomyocytes. These cells are particularly susceptible to free radical damage due to a deficiency in antioxidant enzymes such as catalase (CAT) and superoxide dismutase (SOD) or glutathione peroxidase (GSH-Px) (6, 7).

Cardiac ultrasound or echocardiography is routinely employed to evaluate cardiac function in cardiovascular disease (8). Speckle-tracking echocardiography (STE) is a modern, well-validated, and reproducible method of assessing left ventricular (LV) longitudinal deformation and providing a sensitive assessment of myocardial contractibility (9). Laboratories mainly record LV strain patterns in the long axis and use LV global longitudinal strain (GLS) calculated as the average from all segments, as a measure of global LV function (10).

Traditional Chinese medicine (TCM), with a history of thousands of years of clinical practice, has noticeably attracted the attention of clinicians (11, 12). Hong Huang Decoction (HHD) is a type of TCM composed of *Rhodiola rosea*, astragalus, turmeric, and rhubarb and is able to suppress oxidative stress *in vivo* (13). Flavonoids in traditional Chinese medicine can inhibit inflammatory factors such as IL-6 and reduce inflammatory responses (14). Moreover, Emodin can inhibit the inflammatory response enhancing cholesterol efflux, destroying lipid valves as well as inhibiting proinflammatory factors and chemokines (15). Our previous study revealed that HHD improves cardiac function in breast cancer patients undergoing chemotherapy (16). The composition and efficacy of HHD are shown in Table 1.

**Table 1:** HHD composition.

**Name (botanical, common PinYin names), traditional daily dose (grams)**	**Active compounds**	**Clinical and pharmacological effects**	**Adverse effects/toxicity**
Rhodiola crenulata, Root of Kirilow Rhodiola, Hong jingtian, 3–10 g	Salidroside were detected in Rhodiola (17)	In a clinical study of 60 breast cancer patients, Zhang found that salidroside can provide a protective effect on epirubicin-induced early left ventricular regional systolic dysfunction in patients with breast cancer, and this effect may be induced by antioxidants (18)	No reported adverse events
Rheum Palmatum L., Rhubarb root and rhizome, Da Huang, 3–15 g	Aloe emodin, Rhein. Emodin, chrysophanol are introduced (19)	It has anti-microbial and anti-oxidative effects (20, 21)	The toxicity includes hepatotoxicity and nephrotoxicity. No adverse reactions were observed in mice with dose of 400 mg/kg (22)
Curcuma Longa L., Turmeric, Jianghuang, 3–10 g	The Turmeric contains curcumin (23)	An myocardial injury model in mice (*in vivo*) was established by He et al. the cardioprotective effects of Cur determined by its antioxidation (24)	No reported adverse events
Astragalus Membranaceus (Fisch.) Bge., Astragalus root extract, Huangqi, 9–30 g	Astragaloside iv and Pistil isoflavone glucoside were detected in AM (25)	Anti-inflammatory and anti-oxidative effects was observed in AM (26) As an antioxidant, attenuates DOX-induced cardiomyopathy through the suppression of NADPH oxidase (27)	No reported adverse events

The present study aimed to assess the effects of HHD on myocardial injury in breast cancer patients who had undergone chemotherapy with ANT.

## Methods

### Study design

This randomized placebo-based trial was conducted in accordance with the CONSORT-SPI 2018 checklist. Eligible patients were randomized to either the HHD group or placebo group.

### Study subjects

#### Inclusion criteria

i) Female patients who were diagnosed with breast cancer based on pathology;
ii) Patients aged between 18 and 70 years old;
iii) Normal cardiac function defined as left ventricular ejection fraction (LVEF) > 55% and New York Heart Association (NYHA) functional class I; cardiac troponin levels within the normal range;
iv) Patients who received at least 4 sessions of ANT chemotherapy.

#### Exclusion criteria

i) Patients who were not recommended for chemotherapy owing to various underlying problems;
ii) Patients who participated in other clinical trials;
iii) Pregnant and lactating women;
iv) Patients who did not sign the written informed consent form;
v) Patients with a history of alcohol or drug abuse.

### Interventions

All patients received 4–8 rounds of chemotherapy with ANT. The ANT dose was administered based on the patient's body surface area and on the day of treatment, antacid, and antiemetic drugs (e.g., omeprazole) were routinely given. In addition, guided by results from routine blood tests as well as liver and kidney function tests, other required medications may have been prescribed. The chemotherapy protocols and management of adverse events unrelated to cardiac toxicity were in accordance with the Guidelines of the Chinese Society of Clinical Oncology (CSCO) on Diagnosis and Treatment of Breast Cancer (28).

### Preparation of HHD

HHD was acquired from Jiangsu Provincial Hospital of TCM (Nanjing, China). The total weight of the crude herbs was 56 g. The herbs were blended in 400 mL of double- distilled water (1:8, w/v) for 1 h and heated at 100°C for 2 h. After continuous boiling for 2 h, the sample volume was reduced to 200 mL. Subsequent preparation steps were completed at Jiangsu Provincial Hospital of TCM. Each patient ingested 200 mL HDD as a split dose, twice daily during the whole chemotherapy period. HHD and placebo were provided in liquid form in similar non-transparent bags to ensure the patients were not privy to which treatment was being administered apart from a potential difference in taste.

### Outcome measures

#### Primary outcome measures

Two-dimensional speckle tracking echocardiograph (STE) was performed with the GE Vivid E9 system (General Electric Healthcare, Milwaukee, WI, USA). LVEF was assessed by two-dimensional echocardiography using the modified Simpson's method (29). LVEF was measured at three time points (before chemotherapy, 3 months after the first round of chemotherapy, and 6 months after the first round of chemotherapy).

#### Secondary outcome measures

GLS was measured with the above STE using the GE Vivid E9 system. After LVEF measurements were completed, the sector angle was adjusted and the settings optimized. The apical four-chamber section was selected and continuous three-dimensional dynamic images of four cardiac cycles were collected. The three-dimensional strain speckle tracking analysis software automatically calculated the GLS based on the collected images.

#### Measuring diagnostic markers of acute myocardial infarction

Blood samples (5 ml) were extracted 1 day before undergoing chemotherapy and again at 3 and 6 months after initiation of chemotherapy. The samples were subjected to measurements of diagnostic markers for acute myocardial infarction which were lactate dehydrogenase (LDH), creatine kinase-MB (CK-MB) and B-type natriuretic peptide (BNP).

#### Detection of the levels of inflammatory cytokines

The blood samples were also drawn in blood collection tubes without anticoagulant and sera were prepared by centrifugation at 500 g for 10 min at room temperature. The sera samples were then analyzed for levels of inflammatory cytokines. Serum levels of nitric oxide (NO) and SOD were measured at 550 nm using the Shimadzu-UV-160 spectrophotometer (Shimadzu Corp., Tokyo, Japan) and levels of NO and SOD were calculated. Levels of tumor necrosis factor-α (TNF-α), interleukin-6 (IL-6), interleukin-10 (IL-10) and human C-reaction protein (CRP) in the sera were evaluated by enzyme-linked immunosorbent assay (ELISA; Multisciences (Lianke) Biotech Co. Ltd., Hangzhou, China).

#### Randomization

Patients were randomly assigned to study groups by non-stratified randomization. The random sequence was generated using the random number function of the Microsoft^®^ Excel software. The randomization list was kept on a password-secured computer. All patients, study site personnel, raters, and contract research organization staff members were blinded to the group assignment.

#### Ethical statement and informed consent

The research protocol was approved by the Institutional Review Board of the Affiliated Hospital of Nanjing University of Traditional Chinese Medicine (Nanjing, China; Approval No. 2018nl-164-02). From April 2019 to December 2020, patients who were admitted to the breast cancer department of our hospital were screened and enrolled in the current study. All the eligible patients signed the written informed consent form prior to commencing the study. The research protocol was registered at the Chinese Clinical Trial Registry (Reg. No. ChiCTR1900022394).

### Statistical analysis

Data obtained from the samples collected over different time points were compared using the Student's *t*-test, and differences were compared using the *t*-test or the Student's *t*-test where appropriate, based on the data distribution. Categorical variables were expressed as percentages, and differences were compared using the chi-square or Fisher's exact test, where appropriate, based on the expected counts. All statistical analyses were performed using the SPSS 17.0 software (IBM Corp., Armonk, NY, USA).

## Results

### Patients' baseline demographic and clinical characteristics

**Figure 1:**
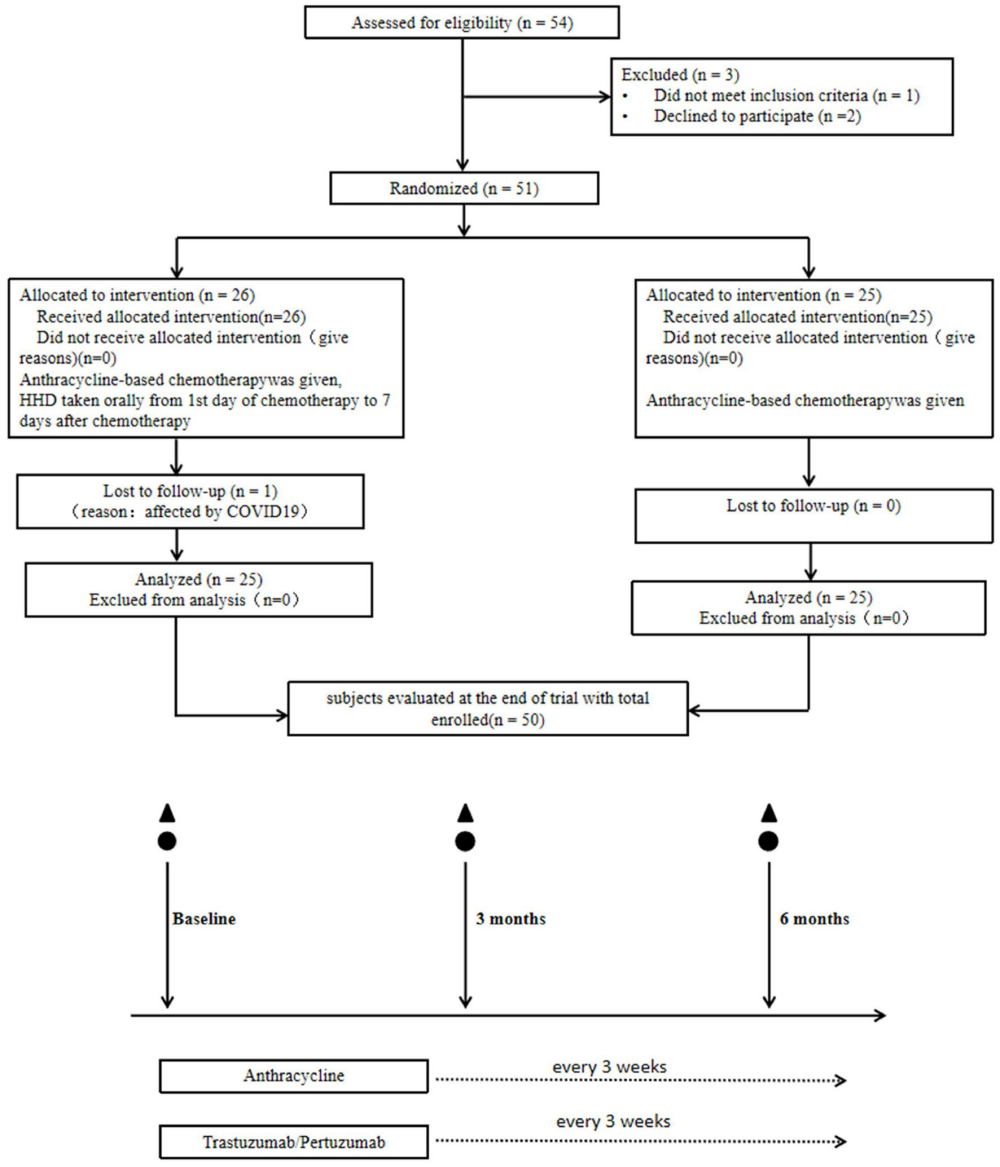
Participant flow through the trial. ▴, echocardiogram with speckle-tracking strain analysis; ⚫, myocardial enzymes, brain natriuretic peptide, oxidative stress, inflammatory factors in plasma measured pre- and post-chemotherapy infusion every 3 months at 0, 3, and 6 months.

A total of 54 patients were assessed for eligibility, of which 51 cases were enrolled as study subjects. Figure 1 shows the flowchart followed for patient selection. Patients' demographic and clinical characteristics at the beginning of the study are presented in Table 2. There was no significant difference in age, type of surgery, neoadjuvant chemotherapy, and chemotherapy regimen between the HHD-administered and placebo groups (*P* > 0.05).

**Table 2:** The general information of recruited patients.

**General data**		**Treatment group**	**Placebo group**	***P*** **-value**
Age (year)		48.5	47.5	0.652
Tumor size	≥2 cm	16	15	0.771
	<2 cm	9	10	
Neoadjuvant chemotherapy		7	8	0.785
Operation mode	^*^SM + SNLB	11	11	0.885
	^*^MRM	11	12	
	^*^BC	3	2	
Lymphatic metastasis (number)	0	13	12	0.943
	1–3	6	6	
	≥4	6	7	
Targeted therapy		4	4	1
Chemotherapy regimens	#EC-T	6	6	0.948
	#EC	9	8	
	#EC_2W_-T	10	11	
Radiotherapy		10	10	1

* *SM + SNLB, simple mastectomy + sentinel lymph node biopsy; MRM, modified radical mastectomy; BC, Breast-conserving surgery*.

^#^*Patients receive either four cycles of EC (Epirubicin 90 mg/m^2^; Cyclophosphamide 600 mg/m^2^, day 1, every 3 weeks), or eight cycles of EC-T (four cycles of Epirubicin 90 mg/m^2^; Cyclophosphamide 600 mg/m^2^, day 1, every 3 weeks, followed by four cycles of docetaxel 75 mg/m^2^, day 1, every 3 weeks), or eight cycles of EC2W-T (four cycles of Epirubicin 90 mg/m^2^; Cyclophosphamide 600 mg/m^2^, day 1, every 2 weeks, followed by four cycles of docetaxel 75 mg/m^2^, day 1, every 2 weeks)*.

### LVEF

The LVEF value for the two groups of patients decreased after undergoing chemotherapy but the difference was not statistically significant (treatment group: *P* = 0.094; placebo group: *P* = 0.060). After 6 months of chemotherapy, the LVEF value did not significantly change (Table 3). This indicated that although the LVEF value could be an important index to evaluate the cumulative ANT cardiotoxicity, our results showed that there was no significant difference in the LVEF value over a short-term (≤6 months), confirming that LVEF would not reveal any early decline in cardiac function.

**Table 3:** Echocardiographic measurements ($\bar{X}$ ± SD) (%).

**Indices**	**Groups**	**Before**	**3 months**		**6 months**	
LVEF	Treatment	66.28 ± 3.02	66.26 ± 2.41	0.970	64.96 ± 2.69	0.094
	Placebo	66.76 ± 1.95	65.68 ± 2.34	0.045	65.48 ± 3.12	0.060
	*P*-value	0.596	0.392		0.531	

*The values in the fifth column mean differences between 3 months after the start of chemotherapy compared to before chemotherapy; the values in the seventh column mean differences between 6 months after the initiation of chemotherapy relative to values before chemotherapy*.

### Echocardiography speckle tracking imaging (GLS)

**Figure 2:**
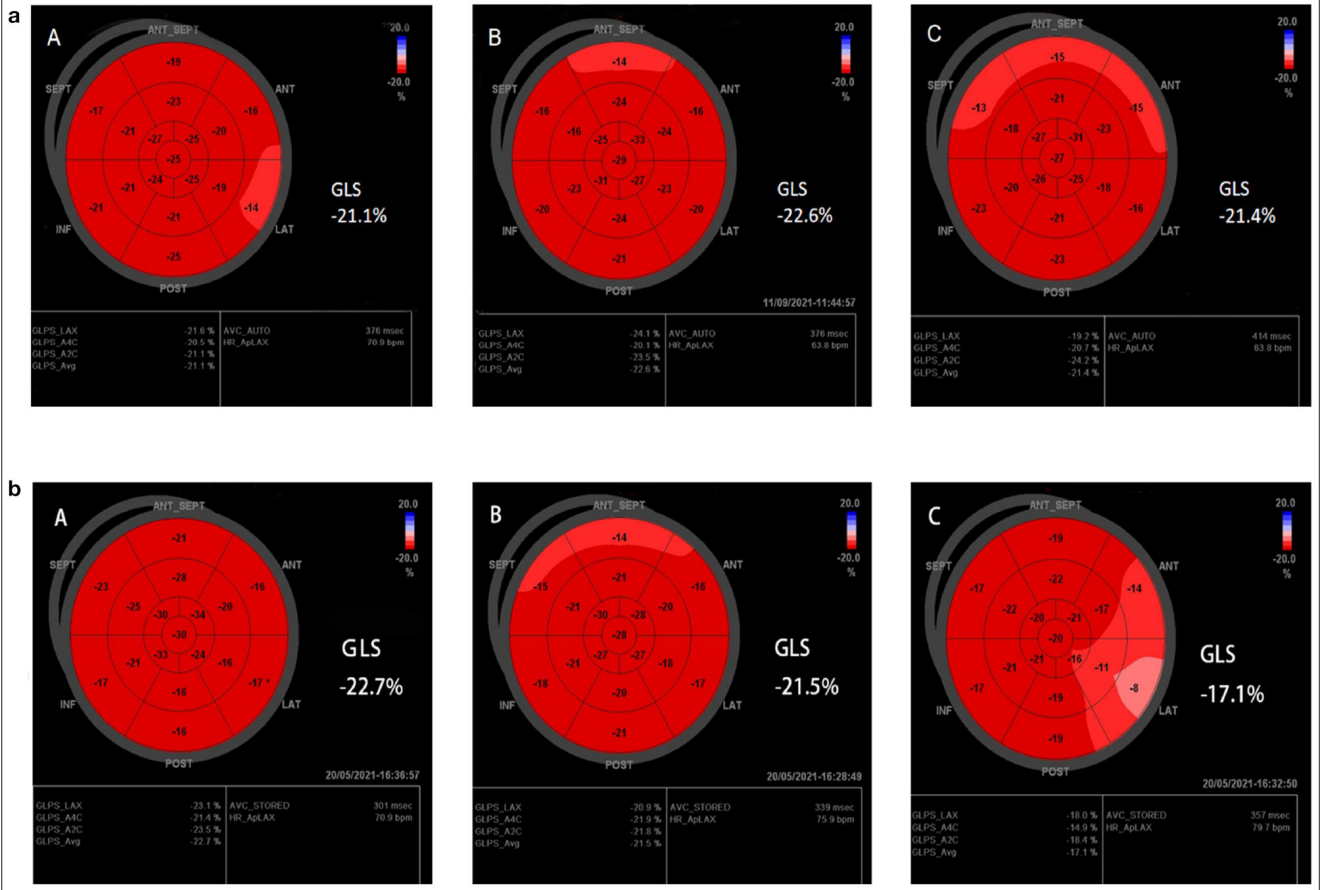
Typical cases. **(a,b)** Speckle-tracking echocardiography of treatment group, and placebo group, respectively. GLS was statistically significant compared with the placebo group after 6 months of HHD treatment. [**(A)** Pre-therapy, **(B)** 3M, **(C)** 6M].

**Figure 3:**
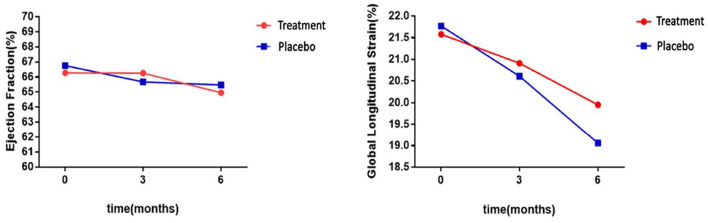
Statistical analyses of LVEF and GLS differences between groups. LVEF levels were not significantly different after 6 months of HHD treatment, but GLS decreased significantly.

**Figure 4:**
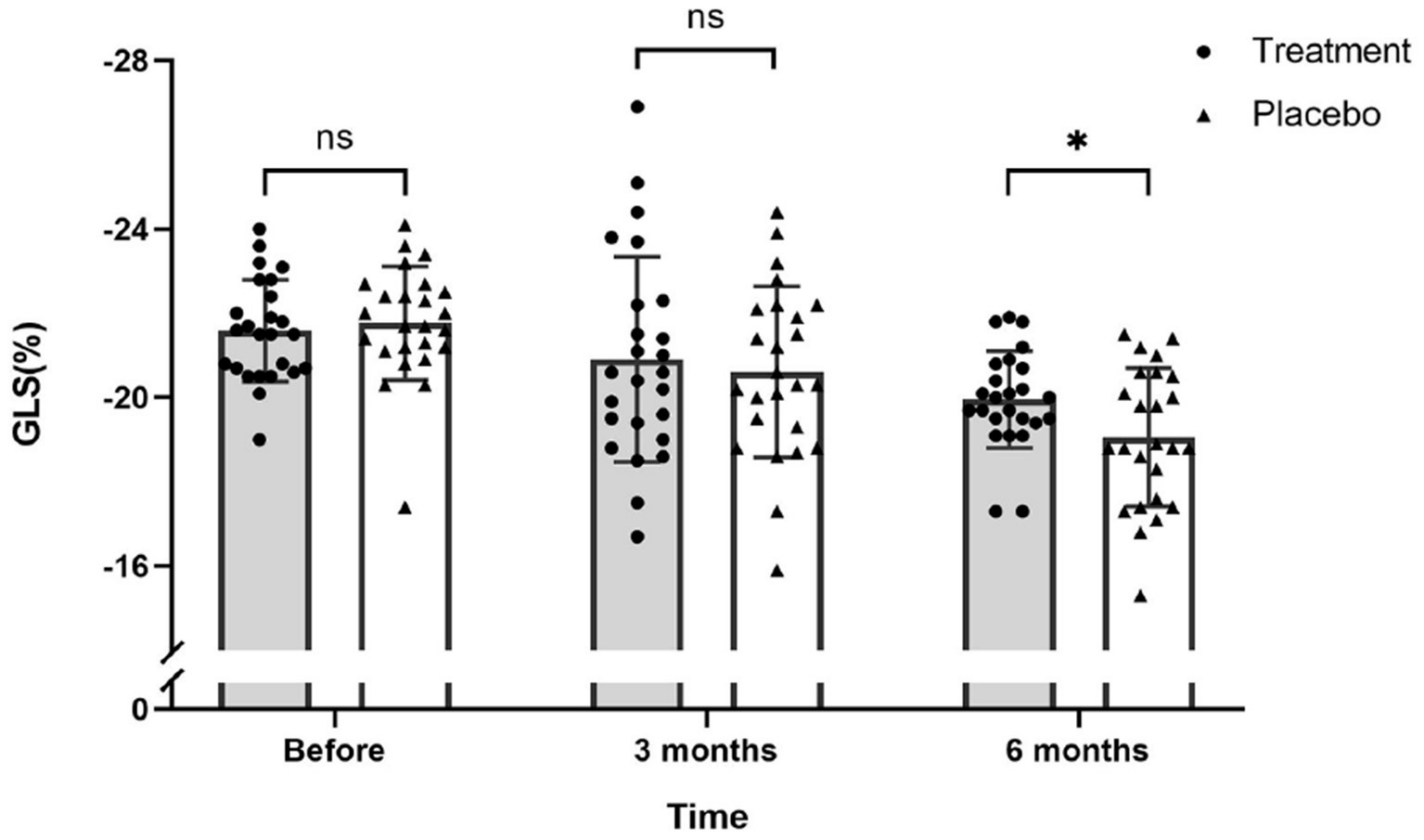
Scatter plot for GLS values of 51 patients on the day before chemotherapy, 3 months after the first round of chemotherapy and 6 months after the first round of chemotherapy. There was no significant difference in GLS values between the treatment and placebo groups before and after 3 months of chemotherapy. After 6 months of chemotherapy, the GLS values of the treatment group was significantly higher than the placebo group (ns, not significant; *P ≤ 0.05).

Interestingly, GLS value of a typical case in the treatment group was significantly higher than the placebo group after 6 months of chemotherapy (−21.4 vs. −17.1%) (Figure 2). At the same time, the GLS values for both treatment and placebo groups decreased after 6 months of chemotherapy compared with the pre-chemotherapy measurement (Figure 3) (treatment group: −21.58 ± 1.21 vs. −19.95 ± 1.16, *P* ≤ 0.001; placebo group: −21.77 ± 1.35 vs. −19.06 ± 1.64, *P* ≤ 0.001; Table 4). This suggests that GLS is a better indicator of cardiotoxicity caused by ANT compared to LVEF (Figure 4). These results showed that HHD had a protective effect on the early treatment of myocardial injury caused by ANT during chemotherapy. The utility of early strain changes to predict subsequent cardiotoxicity is as shown in Figure 2.

**Table 4:** Echocardiography speckle tracking imaging (X ± SD).

**Indices**	**Groups**	**Before**	**3 months**		**6 months**	
GLS	Treatment	−21.58 ± 1.21	−20.91 ± 2.44	0.22	−19.95 ± 1.16	≤0.001
	Placebo	−21.77 ± 1.35	−20.61 ± 2.03	0.003	−19.06 ± 1.64	≤0.001
	*P*-value	0.613	0.638		0.03	

*The values in the fifth column mean differences between 3 months after first chemotherapy and before chemotherapy; the values in the seventh column mean differences between 6 months after first chemotherapy and before chemotherapy*.

### Measurement of diagnostic markers of acute myocardial infarction

**Figure 5:**
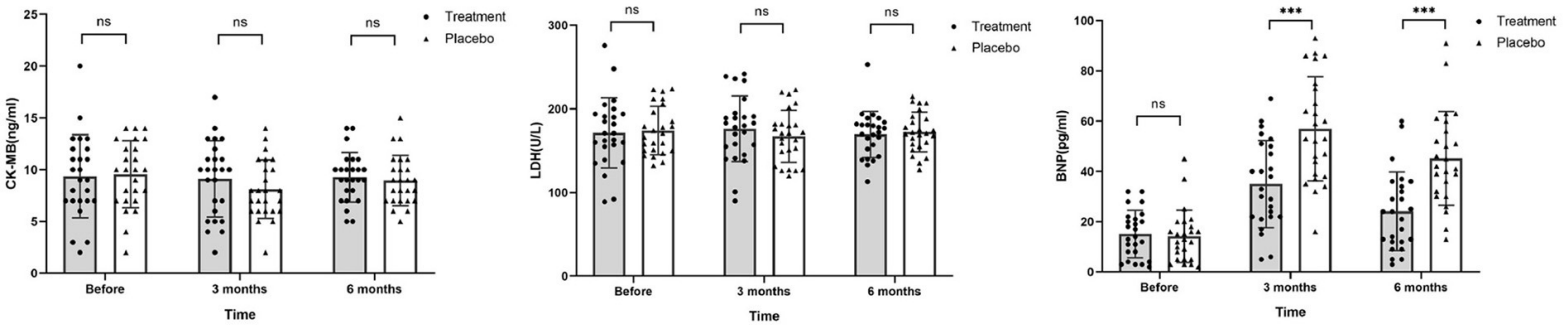
Levels of CK-MB, LDH, and BNP before chemotherapy, 3 and 6 months after chemotherapy. Before and after chemotherapy, the values of CK-MB and LDH in both groups decreased to a certain extent, but the difference was not statistically significant. BNP values in the placebo group were significantly higher than that in the treatment group after chemotherapy (ns, not significant; ***P ≤ 0.001).

After chemotherapy, the plasma levels of CK-MB and LDH in both groups were reduced compared to those recorded before chemotherapy was initiated. In addition, BNP levels increased after 3 months of chemotherapy for both groups (Figure 5). However, levels for the HHD-treatment group were significantly lower than those in the placebo group (P < 0.05, Table 5).

**Table 5:** Myocardial enzyme lists and related hormones (X ± SD).

**Indices**	**Groups**	**Before**	**3 months**		**6 months**	
CK-MB (ng/ml)	Treatment	9.36 ± 4.01	9.12 ± 3.69	0.715	9.28 ± 2.39	0.896
	Placebo	9.56 ± 3.24	8.12 ± 2.82	0.1	8.96 ± 2.42	0.505
	*P*-value	0.847	0.287		0.640	
LDH (U/L)	Treatment	171.40 ± 42.11	176.30 ± 39.24	0.595	169.52 ± 27.60	0.777
	Placebo	174.16 ± 28.88	167.30 ± 31.12	0.461	172.48 ± 23.76	0.840
	*P*-value	0.788	0.371		0.686	
BNP (pg/ml)	Treatment	15.04 ± 9.42	34.96 ± 17.31	≤0.001	24.08 ± 15.63	0.014
	Placebo	14.16 ± 10.40	56.96 ± 20.71	≤0.001	45.20 ± 18.65	≤0.001
	*P*-value	0.692	≤0.001		≤0.001	

*The values in the fifth column mean differences between 3 months after first chemotherapy and before chemotherapy; the values in the seventh column mean differences between 6 months after first chemotherapy and before chemotherapy*.

### The levels of inflammatory cytokines

**Figure 6:**
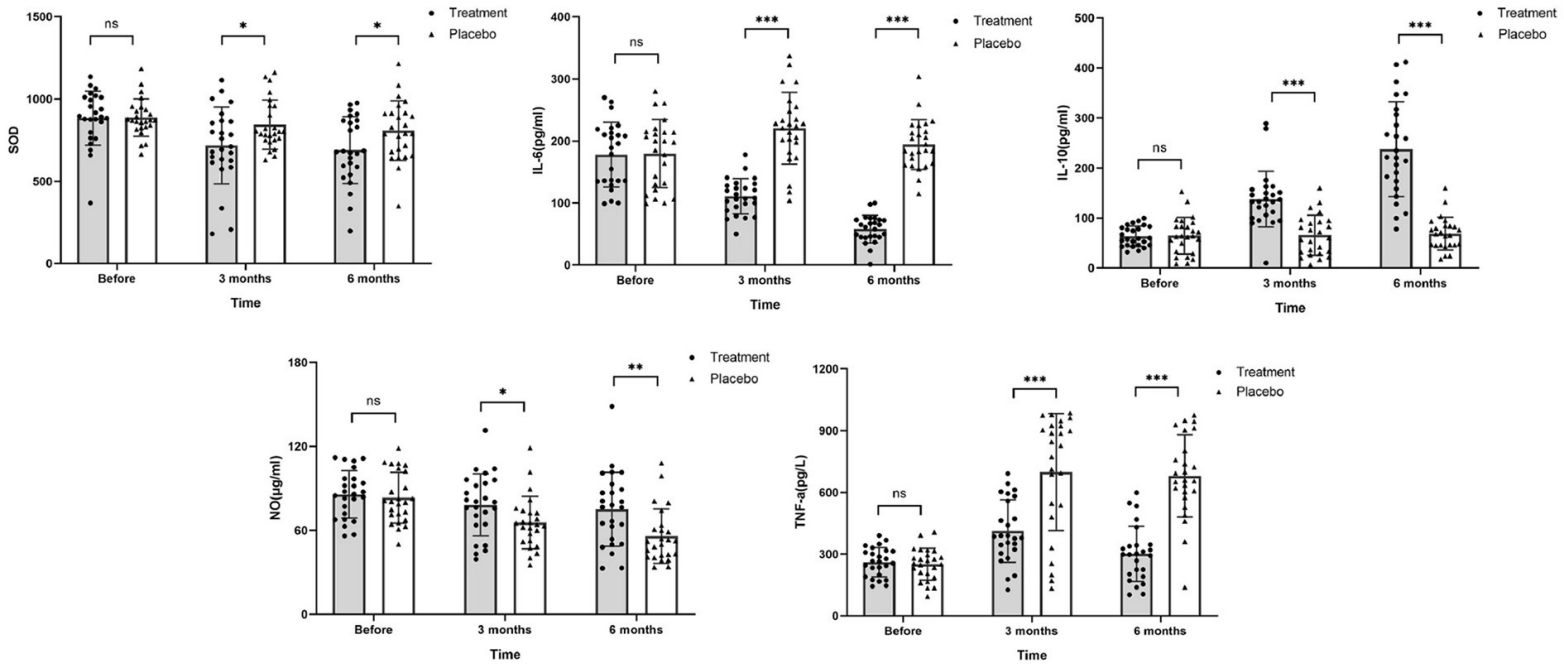
Changes in SOD, IL-6, IL-10, NO, and TNF-α levels after chemotherapy. The values of SOD, IL-6, and TNF-α in the treatment group were higher than those in the placebo group, while levels of IL-10 and NO were noticeably lower than those in the placebo group after chemotherapy (ns, no significance difference; *P ≤ 0.05, **P ≤ 0.01, and ***P ≤ 0.001).

SOD levels decreased to within 100 U/L after 3 months of chemotherapy in the HHD-treated group. On the other hand, NO levels were significantly reduced after chemotherapy in the both groups with the reduction more pronounced in the placebo group than that in the treatment group. After 3 months of chemotherapy, TNF-α levels was elevated to nearly 3 times higher than that before chemotherapy in the placebo group (698.70 ± 284.50 vs. 251.00 ± 77.49, *P* ≤ 0.001), while levels were elevated 1.6 times in the treatment group (412.20 ± 152.10 vs. 261.10 ± 70.55, *P* ≤ 0.001). IL-6 and IL-10 are important indicators of inflammation. After 6 months of chemotherapy, IL-6 levels decreased significantly in the treatment group (58.04 vs. 177.90, *P* < 0.05), whereas IL-10 levels were markedly elevated (237.90 vs. 63.44, *P* < 0.05). The results indicate that HHD provides antioxidant and anti-inflammatory effects on the body during chemotherapy. Before chemotherapy, there was no significant difference in the levels of IL-6 and IL-10 between the two groups. After 6 months of chemotherapy, there was a significant difference in the levels of IL-6 and IL-10 between the two groups (*P* < 0.05; Figure 6). Combined with the changes in GLS in the two groups, HHD may improve the repair of early myocardial injury caused by ANTs during chemotherapy by reducing oxidative stress and inflammation. Further data are listed in Table 6.

**Table 6:** Oxidative stress and inflammatory plasma markers.

**Indices**	**Groups**	**Before**	**3 months**		**6 months**	
SOD	Treatment	883.50 ± 164.26	717.48 ± 233.11	0.003	689.71 ± 203.60	≤0.001
(u/L)	Placebo	887.04 ± 114.26	844.56 ± 150.04	0.228	807.88 ± 182.10	0.109
	*P*-value	0.930	0.026		0.036	
IL-6 (pg/ml)	Treatment	177.90 ± 52.22	110.80 ± 28.57	≤0.001	58.04 ± 22.06	≤0.001
	Placebo	179.60 ± 54.89	220.50 ± 57.8	≤0.001	194.20 ± 40.14	0.171
	*P*-value	0.912	≤0.001		≤0.001	
IL-10 (pg/ml)	Treatment	63.44 ± 20.24	137.60 ± 55.70	≤0.001	237.90 ± 94.98	≤0.001
	Placebo	64.68 ± 36.82	65.64 ± 40.23	0.905	68. ± 32.92	0.581
	*P*-value	0.883	≤0.001		≤0.001	
NO (μmol/L)	Treatment	85.64 ± 16.90	78.18 ± 22.22	0.049	75.05 ± 26.39	0.043
	Placebo	83.22 ± 18.19	65.58 ± 18.73	≤0.001	55.83 ± 19.37	≤0.001
	*P*-value	0.630	0.035		0.005	
TNF-α (pg/L)	Treatment	261.10 ± 70.55	412.20 ± 152.10	≤0.001	301.80 ± 134.20	0.164
	Placebo	251.00 ± 77.49	698.70 ± 284.50	≤0.001	680.30 ± 199.60	≤0.001
	*P*-value	0.634	≤0.001		≤0.001	

*The values in the fifth column mean differences between 3 months after first chemotherapy and before chemotherapy; the values in the seventh column mean differences between 6 months after first chemotherapy and before chemotherapy*.

## Discussion

Cardiotoxicity is a potential complication of breast cancer chemotherapy and attempts to attenuate the complication may lead to the use of reduced dosage and subsequent loss of clinical effectiveness. This in turn would have an impact on patients' morbidity, mortality, and quality of life, which are independent of the original prognosis.

Improved breast cancer survival together with better awareness of the later-stage effects of cardiotoxicity has led to growing recognition for surveillance of ANT- treated cancer survivors with early intervention to treat or prevent heart failure (30). Echocardiography is the most frequently used method to detect cardiac injury in breast cancer patients (31). Our study revealed that for breast cancer patients, LVEF values decreased after 6 months of ANT chemotherapy with no significant difference between post- and pre-chemotherapy. Similarly, treatment with HHD did not provide any significant difference in LVEF values when compared to the placebo group. Moreover, the GLS value was markedly reduced after 6 months of chemotherapy although significantly higher in the HHD treated group compared to the placebo group of patients. Current guidelines recommend the quantification of LVEF before and after chemotherapy with additional scanning for high-risk patients. Studies have shown that cardiac imaging with echocardiographic measurement of GLS can detect myocardial injury in the early stage prior to the development of left ventricular dysfunction (32). Our study also found that GLS had a more noticeable effect than LVEF on ANT-induced myocardial injury in the early stages (within 6 months) of chemotherapy. After 6 months of chemotherapy, breast cancer patients are generally in the early stages of follow-up, and assessment of GLS value during this follow-up period is key to identifying myocardial injury in the patients.

Our results showed that the GLS values of the two groups decreased after 6 months of chemotherapy, especially in the placebo group. HHD improved the GLS value but not to normal levels. This may be a result of insufficient treatment time or patients suffering from subclinical myocardial injury (as indicated by the GLS value) at that stage of follow-up, which had not yet developed into more serious cardiac dysfunction (33).

Early myocardial injury is also related to inflammation caused by IL-6, IL-10, and TNF-α cytokine storm (34–36). After myocardial injury, there are two different inflammatory phases: one is the initial pro-inflammatory phase of eliminating damaged cells, and the other is the anti-inflammatory reparative phase leading to wound healing and scar formation. IL-6 and IL-10 participate in these two respective phases (37). In particular, IL-6 is a valuable biomarker for predicting future adverse events in patients with myocardial injury (38). From our study, it is evident that after 6 months of chemotherapy, the level of pro-inflammatory factor IL-6 decreased significantly and the level of anti-inflammatory factor IL-10 increased significantly in the treatment group. This further validates that HHD has a superior anti-inflammatory property. Combined with the above GLS value, it can be inferred that HHD protects the myocardium by regulating the inflammatory response.

A number of potential cardioprotective drugs have been explored (39). Dexrazoxane is the only drug for patients with advanced breast cancer. Dexrazoxane's cardioprotective mechanism against ANT was proposed to be due to iron chelation, preventing ANT-iron binding and ROS generation. Angiotensin-converting enzyme (ACE)-inhibitors, angiotensin-receptor blockers (ARBs) and beta-blockers have also been used to prevent ANT cardiotoxicity (2, 4). A meta-analysis showed that there was no robust evidence to support the routine use of cardiac protective agents or liposomal formulations. The study also highlighted that there was a need to improve cardiac monitoring during oncology trials (40). Captopril and carvedilol are commonly used for the assessment of cardiotoxicity in clinical trials, however, their effects have not been fully explored (41). Our study has shown that HHD had a cardioprotective effect on early-stage breast cancer patients.

ANT cardiotoxicity is associated with DNA damage, inhibition of protein synthesis, mitochondrial biogenesis, induction of apoptosis, inflammation, and generation of ROS (5, 42). Nicotinamide adenine dinucleotide phosphate (NADPH) oxidase-2 (NOX2) is abundant in inflammatory cells, where it is suggested to contribute to oxidative stress (43). The NOX2 generating ROS promotes the expression of inflammatory cytokines. Oxidative stress is associated with the process of inflammation. Oxidation aggravates the inflammatory reaction, and inflammation promotes oxidation through inflammatory mediators (44–47).

Studies have confirmed that TCM can regulate the state of oxidative stress and inflammation and hence, regulate and reduce ANT cardiotoxicity. Curcumin is an anti-oxidant (48) while Salidroside in Rhodiola can regulate SOD levels and protect Lipopolysaccharide-induced myocardial infarction through the ROS mediated PI3K/Akt/mTOR signaling pathway. This indicates that Salidroside in Rhodiola can protect cardiomyocytes through anti-oxidation (49), and salidroside can mediate TNF-α MAPK and NF-κB activation in induced cardiac microvascular endothelial cells (CMECs) to reduce the inflammation of heart and blood vessels (50). This is also reflected by the changes for inflammatory factors IL-6, IL-10, and TNF-α observed in our study (Figure 6).

In summary, our study showed that HHD could regulate the levels of IL-6, IL-10, SOD, NO, and TNF-α and GLS was a better indicator of early-stage myocardial injury triggered by ANT. The results also suggest that HHD could modulate oxidative stress to protect against ANT cardiotoxicity.

## Data availability statement

The data presented in the study are deposited in the Resman IPD, accession number ChiCTR1900022394.

## Ethics statement

The studies involving human participants were reviewed and approved by Ethics Committee of the Affiliated Hospital of Nanjing University of traditional Chinese medicine (Jiangsu Hospital of traditional Chinese Medicine). The patients/participants provided their written informed consent to participate in this study.

## Author contributions

SC and JX contributed to conception and design of the study and wrote the first draft of the manuscript. SC, JX, LC, and YH carried out case collection and organized the database. ML and JZ conducted outcome measures. MF and HW performed the statistical analysis. LC and YH wrote sections of the manuscript. CY gave academic and technical guidance. All authors contributed to manuscript revision, read, and approved the submitted version.

## Funding

This study was supported by a program funded by the National Natural Science Foundation of China (Grant No. 81873305), the peak of academic talent project of Jiangsu Provincial Hospital of traditional Chinese medicine (Grant No. Y2021rc09), a grant from the Natural Science Foundation Project of Jiangsu Province (Grant No. SBK2019042518), and as well as the Postgraduate Research & Practice Innovation Program of Jiangsu Province (Grant No. KYCX22_1921).

## Conflict of interest

The authors declare that the research was conducted in the absence of any commercial or financial relationships that could be construed as a potential conflict of interest.

## Publisher's note

All claims expressed in this article are solely those of the authors and do not necessarily represent those of their affiliated organizations, or those of the publisher, the editors and the reviewers. Any product that may be evaluated in this article, or claim that may be made by its manufacturer, is not guaranteed or endorsed by the publisher.
